# Analysis of clinical characteristics and factors affecting treatment responses in patients with pyoderma gangrenosum: a multicenter study of 239 patients^☆^^☆^

**DOI:** 10.1016/j.abd.2024.02.002

**Published:** 2024-05-11

**Authors:** Funda Erduran, Esra Adışen, Yıldız Hayran, Güneş Gür Aksoy, Erkan Alpsoy, Leyla Baykal Selçuk, Sibel Doğan Günaydın, Ayça Cordan Yazıcı, Ayşe Öktem, Malik Güngör, Elif Afacan, Deniz Devrim Kuşçu, Leyla Elmas, Kübra Aydoğan, Dilek Bayramgürler, Evren Odyakmaz Demirsoy, Melih Akyol, Rukiye Yasak Güner, Hilal Kaya Erdoğan, Ersoy Acer, Tulin Ergun, Savaş Yaylı, Ferhan Bulut, Esra Saraç, Akın Aktaş

**Affiliations:** a Department of Dermatology, Ankara Bilkent City Hospital, Ankara, Turkey; b Department of Dermatology, Gazi University Faculty of Medicine, Ankara, Turkey; c Department of Dermatology, Akdeniz University Faculty of Medicine, Antalya, Turkey; d Department of Dermatology, Karadeniz Technical University Faculty of Medicine, Trabzon, Turkey; e Department of Dermatology, Hacettepe University Faculty of Medicine, Ankara, Turkey; f Department of Dermatology, Mersin University Faculty of Medicine, Mersin, Turkey; g Ankara University Faculty of Medicine, Department of Dermatology, Ankara, Turkey; h Department of Dermatology, Kocaeli University Faculty of Medicine, Kocaeli, Turkey; i Department of Dermatology, Sivas Cumhuriyet University Faculty of Medicine, Sivas, Turkey; j Department of Dermatology, Eskişehir Osmangazi University Faculty of Medicine, Eskişehir, Turkey; k Marmara University Faculty of Medicine, Department of Dermatology, İstanbul, Turkey; l Department of Dermatology, Koç University Faculty of Medicine, İstanbul, Turkey

**Keywords:** Comorbidity, Hereditary autoinflammatory diseases, Immunosuppressive agents, Pyoderma gangrenosum, Regression analysis

## Abstract

**Background:**

Pyoderma Gangrenosum (PG) is a chronic disease characterized by recalcitrant skin ulcers.

**Objective:**

We aimed to evaluate the demographic, clinical characteristics, treatments and factors affecting the treatment responses of patients with PG.

**Methods:**

We performed a multicenter study of 12 tertiary care centers. We analyzed the data of the patients who were followed up with a diagnosis of PG between the years 2012‒2022 retrospectively.

**Results:**

We included a total of 239 patients of whom 143 were female and 96 were male, with an average age of 54.2 ± 17.4 years. The most common treatment was systemic steroids (n = 181, 75.7%). Among these patients, 50.8% (n = 92) used systemic steroids as the sole systemic agent, while 49.2% (n = 89) used at least one adjuvant immunosuppressive agent. The independent factors determined in regression analysis to influence response to systemic steroids positively were disease onset age ≥ 30-years, negative pathergy, absence of leukocytosis, negative wound culture, presence of a single lesion, and absence of upper extremity involvement. Biological agents were used in 18.4% (n = 44) of the patients in the present study. We also analyzed pathergy positive PG and early onset (onset age < 30) PG separately due to their distinct clinical features which were revealed during statistical analysis.

**Study limitations:**

Retrospective nature of the present study.

**Conclusions:**

Analyses of the factors influencing treatment responses are addressed in this study. Also, we concluded that investigation for accompanying autoinflammatory diseases of pathergy positive PG and early onset PG is necessary and the patients in these two groups are more resistant to treatment, necessitating more complicated treatments.

## Introduction

Pyoderma gangrenosum (PG) is a chronic disease characterized by recurrent skin ulcers. It was first described by Brocq^1^ and named by Brunsting et al.^2^ Contrary to its name, it was later understood that it's a neutrophilic dermatosis with aseptic neutrophilic infiltration.^3^ The worldwide incidence of PG is estimated to be 3‒10/1000.000.^4^

In its etiopathogenesis, neutrophil dysfunction, clonal T-cell expansion, inflammatory mediators such as Interleukin (IL)-17, IL-23 and genetic predisposition are thought to play a role.^5^ PG has four primary subgroups: classical (ulcerative), bullous, pustular, and vegetative. Rarer variants include peristomal PG, post-operative PG, and drug-induced PG.^6^ In classical PG, which is the most common type, lesions begin as sterile pustules and erythematous papules and transform into painful ulcerative lesions. Lesions with violaceous appearance and sharply defined undermined edges are typical for classical type PG. Lesions heal with cribriform or sieve-like atrophic scars.^7^, ^8^ Pathergy, an exaggerated response following minor skin trauma, is observed in approximately one-third of the patients.^6^ Since laboratory investigations and histopathological findings are not distinctive in PG, the diagnosis is made by excluding other inflammatory skin diseases causing ulceration.^7^ Su et al. suggested diagnostic criteria for PG in 2004.^9^ According to the recently defined Delphi consensus, the presence of neutrophil infiltration in a biopsy specimen taken from the edge of the ulcer is a major criterion that must be fulfilled.^10^ PG can be associated with other conditions in 50%‒75% of patients.^7^ The most common associations are inflammatory bowel diseases (IBD), arthritis, and hematologic disorders. Moreover, PG can be associated with syndromes such as PAPA (pyogenic arthritis, PG, acne), PASH (PG, acne, hidradenitis suppurativa [HS]), PASS (PG, acne conglobata, HS, seropositive spondyloarthropathies), PAPASH (pyogenic arthritis, PG, acne, HS), and PsAPASH (psoriatic arthritis, pyoderma gangrenosum, acne, HS).^7^ There is no gold standard for PG treatment. Treatment should be structured according to the number of lesions, size, location, presence of associated diseases, and the patient's comorbidities. The fundamental pillars of treatment include avoiding triggers, proper wound care, pain control, topical, systemic, and targeted immunomodulatory therapies.^5^, ^6^

In the present study, we aimed to evaluate the demographic, and clinical features of patients with PG as well as factors affecting treatment preferences and treatment responses.

## Materials and methods

We performed a multicenter study which comprised 12 tertiary care centers from 8 different provinces in Turkey. We evaluated retrospectively the patients who were followed up between the years 2012‒2022 with the diagnosis of PG according to the diagnostic criteria suggested by Su et al.^9^ in which there should be fulfillment of 2 major criteria (rapidly progressing painful necrotizing ulcers with irregular violaceous-red undermined borders and exclusion of other causes of skin ulceration) and 2 minor criteria (ulcer development at the site of minor trauma suggesting the presence of pathergy or clinical findings of cribriform scarring, presence of systemic disease associated with PG, histopathological finding of sterile neutrophil infiltration in the dermis, rapid response to systemic steroid therapy). We compiled data from all of the patients with PG who were over 18 years old from the study centers.

Ethics committee approval numbered E1-22-2782 was obtained from Ankara Bilkent City Hospital before the study (17.08.2022). The present study was conducted in accordance with the ethical principles in the Helsinki Declaration.

For all treatments, wound healing of ≥75% as an outcome parameter was evaluated as a “good response”. Benefit less than 75% is evaluated as a “poor-moderate response” in this study.

### Statistical analysis

Data were analyzed using SPSS/IBM for windows 23.0 (Chicago, IL, USA). Descriptive statistics such as number and percentage for categorical variables and mean, median, standard deviation, and Interquartile Range (IQR) for numerical variables were used to describe the sample. Chi-Square significance test or Fisher's Exact test was used for comparing categorical variables, and Student's *t*-test or Mann-Whitney *U* test was used for comparing numerical variables. Pearson and Spearman’s correlation tests were used to evaluate the correlation between two numerical variables. Variables with p-values > 0.2 were further evaluated with multivariate logistic regression analysis to identify independent predictors for treatment response. Model fit was assessed with the Hosmer-Lemeshow test. The statistical significance level was determined as p < 0.05 for all analyses.

During the explorative analysis, we discovered that pathergy-positive PG patients and PG patients with onset age < 30 years had distinct clinical features and treatment responses that were significant. The choice of the 30-year age cut-off was based on both bibliographic (the age defined as the theoretical cut point between emerging adulthood and established adulthood)^11^, ^12^ and evidence-based (best potential in predicting multiple disease, patient and outcome-specific endpoints using statistical inference) considerations. We opted for referring to PG that begins under the age of 30 years as early onset PG and ≥ 30 years as late-onset PG) in this study.

## Results

This study included 143 (59.8%) women and 96 (40.2%) men, a total of 239 patients. The average age of the patients was 54.2 ± 17.4 years. The average disease onset age was 48.2 ± 18.1 years. The disease duration was determined as a median value of 24 (IQR 6‒84) (min‒max: 1‒366) months. The demographic data of the patients and hospitalization information are shown in Table 1. The characteristics of the lesions observed in the patients are shown in Table 2. The number of patients with ulcerative type PG was 203 (85%), which constituted the utmost part of the subtypes. Pyoderma gangrenosum subtypes observed in the patients are shown in Table 2.

**Table 1:** Demographic characteristics of patients and hospitalization information.

Total, n (%)		239		
Female		143 (59.8)		
Male		96 (40.2)		
Mean Age ± SD (years)		54.2 ± 17.4		
Onset Age ± SD (years)		48.2 ± 18.1		
Disease Duration (months) (IQR)		24 ( 6‒84)		
Hospitalization Rate, n (%)	187 (78.2)	Number of hospitalization	Once	89 (37.2)
			Twice	46 (19.2)
			Three times	23 (9.6)
			≥ Four times	29 (12.2)
Total Hospital Stay (day) ‒ Median (IQR) (min‒max)		20 (12‒39) (min‒max: 1‒113)		
Shortest Hospital Stay (day) ‒ Median (IQR)		10 (6‒14)		
Longest Hospital Stay (day) ‒ Median (IQR)		15.5 (10‒23)		

SD, Standart Deviation; IQR, Interquartile range.

**Table 2:** Data related to characteristics of pyoderma gangrenosum.

**Lesion localizations**	**n (%)**
Lower Extremities	189 (79.1)
Upper Extremities	31 (13)
Body	70 (29.3)
Head-neck	8 (3.3)
Other (oral mucosa, genital region, buttocks)	**5 (2.1)**
Largest Lesion Size (cm)	**n (%)**
1‒5	104 (43.5)
5‒10	86 (36)
10‒15	29 (12.1)
15‒20	12 (5)
> 20	8 (3.3)
**Number of Lesions**	n (%)
1 lesion	90 (37.7)
2‒5 lesions	108 (45.2)
5‒10 lesions	30 (12.6)
> 10 lesions	11 (4.6)
**Pyoderma Gangrenosum Types**	n (%)
Ulcerative	203 (84.9)
Bullous	10 (4.2)
Pustular	9 (3.8)
Postoperative	9 (3.8)
Vegetative	5 (2.1)
Peristomal	4 (1.7)
**Comorbidities Associated with Pyoderma Gangrenosum**	n (%)
Inflammatory Bowel Disease	38 (15.9)
Inflammatory Arthritis (Rheumatoid Arthritis, Psoriatic Arthritis, Gout Arthritis)	45 (18.8)
Hematological Diseases	16 (6.7)
Hidradenitis Suppurativa	16 (6.7)
Solid Malignancy	14 (5.9)
Autoinflammatory Diseases (PAPA, PASH, PAPASH)	9 (3.8)
Other (Behçet's Disease)	9 (3.8)
Total	51.5^a^

a Some patients had multiple comorbidities associated with PG.

### Comorbidities associated with PG

The number of patients with at least 1 comorbidity associated with PG was 123 (51.5%). The disease onset age was earlier in patients with PG-related comorbidity compared to those without comorbidity (45.9 vs. 50.7 years, p = 0.048). Inflammatory arthritis was observed in 45 (18.8%) patients. IBD was found in 38 (15.9%) patients. HS was present in 17 (7.1%) patients. The frequency of hematological disease was 6.7% (n = 16). Hematological diseases observed in patients were MGUS (monoclonal gammopathy of undetermined significance), myelodysplastic syndrome, lymphoma, and leukemia. Solid malignancy was present in 14 (5.9%) patients. Autoinflammatory syndromes were found in 9 (3.8%) patients (PAPA, PASH, PAPASH). In patients with the autoinflammatory disease, the frequency of male gender, family history of autoinflammatory disease, multiple region involvement and upper extremity involvement were higher as well as the presence of a higher diameter of the largest lesion and higher number of hospitalizations compared to those without autoinflammatory disease (p-values respectively 0.019, < 0.001, < 0.001, 0.004, 0.006, 0.025). Additionally, these patients were younger, and their disease onset ages were smaller (both p < 0.001). Comorbidities associated with PG are shown in Table 2.

Pathergy was detected in 55 (23%) patients while was negative in 140 (58.6%). In 44 (18.4%) patients, the pathergy status was unknown. Pain was present in 218 (91.2%) patients. Laboratory and histopathological findings of the patients are shown in Table 3.

**Table 3:** Patients' laboratory data and other disease-related data.

**Laboratory Findings**	**n (%)**
Anemia	124 (51.9)
Leukocytosis	93 (38.9)
Neutrophilia	104 (43.5)
Elevated CRP^a^ level	166 (69.7)
Elevated Erythrocyte Sedimentation Rate	53 (22.2)
Electrolyte Disturbance	13 (5.4)
Liver Function Test Abnormality	7 (2.9)
Positive Wound Culture	111 (46.4)
**Histopathological Assessment**	**n (%)**
Compatible with PG	212 (88.7)
Incompatible with PG	8 (3.3)
Biopsy Not Taken	19 (7.9)
**Pathergy Phenomenon**	**n (%)**
Pathergy positive	55 (23)
Pathergy negative	140 (58.6)
Pathergy Status Unknown	44 (18.4)
**Presence of Pain**	**n (%)**
	218 (91.2)

CRP, C-Reactive Protein; PG, Pyoderma Gangrenosum.

### Treatments received by the patients

Wound care was administered to all of the patients. The most frequently used topical treatments were steroids (n = 163, 68.2%), synthetic wound dressings (n = 73, 30.5%), and tacrolimus (n = 40, 16.7%). Since the majority of patients receiving topical treatment also received systemic treatment, responses to topical treatments could not be evaluated in this study.

The average time between diagnosis and the initiation of systemic treatment was 1 month (IQR 0.5‒3).

### Systemic treatments

Total number of the patients who used systemic steroids was 181 (75.7%). Of those patients, 92 (50.8%) used systemic steroids as a single systemic agent, while 89 (49.2%) used at least one immunosuppressive agent in addition to steroids. Characteristics of these two groups are compared in Table 4.

**Table 4:** Comparison of characteristics between patients receiving systemic steroid treatment alone and systemic steroid + additional immunosuppressive treatment.

Characteristics	Systemic steroid, n (%)	Systemic steroid + additional immunosuppressive treatment, n (%)	p
Number of the areas involved			<0.001
Single	81 (88)	56 (62.9)	
Multiple	11 (12)	33 (37.1)	
Number of lesions			<0.001
Single	43 (46.7)	19 (21.3)	
Multiple	49 (53.3)	70 (78.7)	
Presence of lower extremity involvement			0.009
Present	69 (75)	80 (89.9)	
Absent	23 (25)	9 (10.1)	
Wound Culture			0.014
Positive	39 (42.4)	54 (60.7)	
Negative	53 (57.6)	35 (39.3)	
Pathergy			0.018
Positive	16 (17.4)	29 (32.6)	
Negative	76 (82.6)	60 (67.4)	
Leukocytosis			0.032
Present	33 (35.9)	46 (51.7)	
Absent	59 (64.1)	43 (48.3)	
Autoinflammatory Disease			0.003
Present	0 (0)	8 (9)	
Absent	91 (100)	81 (91)	
Inflammatory Bowel Disease			0.040
Present	11 (12)	21 (23.6)	
Absent	81 (88)	68 (76.4)	
Largest Lesion Size			0.008
<5 cm	49 (53.3)	30 (33.7)	
≥5 cm	43 (46.7)	59 (66.3)	
Age, mean (SD) (year)	56.5 (17)	48.7 (15.8)	0.002
Age of Disease Onset (SD) (year)			
<30	9 (9.8)	26 (29.2)	
≥30	83 (90.2)	63 (70.8)	0.001
Disease Duration (month), median (IQR)	12 (3‒50)	60 (24‒120)	<0.001

%, valid percent; SD, Standart Deviation; IQR, Interquartile range.

The median steroid dose was 48 mg (IQR 40‒60) of methylprednisolone-equivalent. The median treatment duration was 60 days (IQR 30‒100). No correlation was found between the steroid dose and the benefit (p = 0.71). A good response to treatment was obtained in 46.4% of the patients. The time interval between diagnosis and the initiation of systemic treatment correlated reversely with benefit from treatment (p = 0.005, *r* = -0.21).

Univariate analysis of characteristics of the patients who responded well to systemic steroid treatment are shown in Table 5. A further multivariate logistic regression analysis was performed (Table 6). Absence of upper extremity involvement (p = 0.041), presence of a single lesion (p = 0.019), disease onset age ≥ 30 (p = 0.001), absence of leukocytosis (p = 0.012), negative wound culture (p = 0.002), negative pathergy (p = 0.005) were determined as the independent factors influencing responses to systemic steroid treatment in multivariate analysis.

**Table 5:** Univariate analysis of patient characteristics with good response or poor-moderate response to systemic steroids.

Characteristics	a Good response, n (%)	a Poor-moderate response, n (%)	p
Number of Regions involved			<0.001
Single	74 (88.1)	63 (64.9)	
Multiple	10 (11.9)	34 (35.1)	
Number of lesions			0.001
Single	39 (46.4)	23 (23.7)	
Multiple	45 (53.6)	74 (76.3)	
Upper Extremity Involvement			0.031
Present	7 (8.3)	19 (19.6)	
Absent	77 (91.7)	78 (80.4)	
Wound Culture			0.006
Positive	34 (40.5)	59 (60.8)	
Negative	50 (59.5)	38 (39.2)	
Pathergy			0.002
Positive	12 (14.3)	33 (34)	
Negative	72 (85.7)	64 (66)	
Leukocytosis			0.001
Present	26 (31)	53 (54.6)	
Absent	58 (69)	44 (45.4)	
Autoinflammatory Disease			0.007
Present	0 (0)	8 (8.2)	
Absent	84 (100)	89 (91.8)	
Presence of Inflammatory Arthritis			0.012
Present	8 (9.5)	23 (23.7)	
Absent	76 (90.5)	74 (76.3)	
Size of the biggest lesion			0.002
<5 cm	47 (56)	32 (33)	
≥5 cm	37 (44)	65 (67)	
Age of Disease Onset (year)			0.002
<30	8 (9.5)	27 (27.8)	
≥30	76 (90.5)	70 (72.2)	
Disease Duration, month			<0.001
<100	76 (91.6)	67 (69.1)	
≥100	7 (8.4)	30 (30.9)	

a Benefit less than 75% is evaluated as poor-moderate response, 75% and above is evaluated as good response.

**Table 6:** Multivariate logistic regression analysis of the characteristics of patients with good response to systemic steroids.

Characteristics of the patients with good response to systemic steroids	p-value	RR	95% CI for RR	
			Lower	Upper
Absence of upper extremity involvement	0.041	3.14	1.05	9.37
Presence of single lesion	0.019	2.39	1.15	4.95
Disease onset age ≥ 30	0.001	6.16	2.19	17.29
Absence of leukocytosis	0.012	2.45	1.22	4.93
Negative wound culture	0.002	3.28	1.58	6.84
Negative Pathergy	0.005	3.55	1.46	8.66

RR, Relative Risk; CI, Confidence Interval.

Number of the patients who used cyclosporine was 85 (35.6%). The median dose of cyclosporine was 300 mg/day (IQR 237.5‒300). The average treatment duration was 100 days (60‒200). Complete recovery was observed in 19 (22.4%) patients. No correlation was seen between the dose of cyclosporine and the benefit (p = 0.61).

Ten patients received intravenous immunoglobulin (IVIG) treatment. Three of these patients achieved a good response.

The conventional systemic treatments and IVIG received by the patients and benefit statuses regarding them are shown in Table 7. As some of the patients used more than one systemic conventional treatment or IVIG concurrently, we could not make a head-to-head comparison in terms of efficacy between these treatments.

**Table 7:** Data related to conventional treatments and IVIG (intravenous immunoglobulin) used by patients included in the study.

	Systemic steroids	Cyclosporine	Azathioprine	Dapsone	Colchicine	IVIG	Methotrexate	Mycophenolate Mofetil	Thalidomide
Number of patients using, n (%)	181 (75.7)	85 (35.6)	30 (12.6)	19 (7.9)	17 (7.1)	10 (4.2)	9 (3.8)	6 (2.5)	1 (0.4)
Dose, median (IQR^a^)	48 mg/day ( 40‒60)	300 mg/day ( 237.5‒300)	150 mg/day (100‒150)	150 mg/day (100‒150)	1.5 mg/day (1‒1.5)	2 g/kg/ 4 weeks	15 mg/week (13.75‒15)	1.5 g/day (1.38‒2)	100 mg/day (100‒100)
Duration, median (IQR)	60 days ( 30‒100)	100 days (60‒200)	360 days (180‒605)	180 days (120‒360)	360 days (200‒990)	1‒3 months	120 days (102‒375)	140 days (75‒200)	200 days (200‒200)
Benefit, n (%)									1 (100)
< 25%	21 (11.6)	10 (11.8)	3 (10)	3 (15.8)	4 (23.5)	3 (30)	3 (33.3)	3 (50)	
25%‒50%	37 (20.4)	19 (22.4)	10 (33.3)	4 (21.1)	9 (52.9)	4 (40)	4 (44.4)	3 (50)	
50%‒< 75%	39 (21.5)	9 (10.6)	9 (30)	2 (10.5)	3 (17.6)	0	1 (11.1)	0	
≥ 75%	32 (17.7)	28 (32.9)	3 (10)	10 (52.6)	0	2 (20)	1 (11.1)	0	
Complete recovery	52 (28.7)	19 (22.4)	5 (16.7)	0	1 (5.9)	1 (10)	0	0	

IQR, Interquartile Range.

a Some of the patients administered more than one systemic conventional treatment concurrently.

### Biological treatments

Biological agents were used in 44 (18.4%) patients. Usage of biological agents was more common in males compared to females (p = 0.005), in patients with a family history of autoinflammatory diseases (p = 0.031) and those with multi-region involvement (p < 0.001), head-neck involvement (p = 0.019), multiple lesions (p = 0.024), comorbidities (p = 0.002), positive wound culture (p = 0.011), positive pathergy (p = 0.006), IBD (p < 0.001), HS (p = 0.012), autoinflammatory diseases (p < 0.001). Patients using biological treatments were younger (p = 0.006), had an earlier onset age (p < 0.001), had more lesions (p = 0.006), more hospital admissions (p = 0.001) and longer hospital stay durations (p = 0.001) compared to those not using biological treatments. The median duration between conventional treatment and biological treatment was 24 months (IQR 12‒60). The biological agents received by the patients and the related response data are shown in Table 8.

**Table 8:** Biological agent treatment data and responses of the patients.

	Number of patients receiving treatment, n (%)	Average dose	Average duration of treatment (months) (IQR)	Treatment Benefit Rates, n %				
				< 25%	25%‒50%	51%‒< 75%	≥ 75%	100%
Infliximab	36 (15.1)	R^a^	12 (7‒22)	2 (5.6)	6 (16.7)	5 (13.9)	11 (30.6)	12 (33.3)
Adalimumab	19 (7.9)	R (42.1%)	14 (12‒27)	6 (31.6)	4 (21.1)	2 (10.5)	2 (10.5)	5 (26.3)
		40 mg/week (47.4%)						
		80 mg/week (10.6%)						
Ustekinumab	4 (1.7)	R	9	1 (25)	3 (75)	0	0	0
Etanercept	3 (1.3)	R	8	1 (33)	2 (67)	0	0	0
Secukinumab	3 (1.3)	R	15	0	2 (67)	0	1 (33)	0
Anakinra	3 (1.3)	100 mg/day	30	1 (33.3)	0	1 (33.3)	1 (33.3)	0
Risankizumab	1 (0.4)	R	4				1 (100)	
Ixekizumab	1 (0.4)	R	6		1 (100)			
Canakinumab	1 (0.4)	300 mg/4-weeks	36				1 (100)	

IQR, Interquartile Range.

a R, Licensed dose.

We additionally analyzed pathergy positive/negative groups and PG patients with onset age < 30/ onset age ≥ 30 which we referred to as early and late-onset PG in the present study. The characteristics of patients with positive and negative pathergy are shown in Table 9. The characteristics of early-onset PG patients are displayed in Table 10.

**Table 9:** Comparison of the characteristics of PG patients with positive and negative pathergy.

Characteristics	Pathergy positive (n = 55)	Pathergy negative (n = 184)	p
Sex, n (%)			0.064
Female	27 (49.1)	116 (63)	
Male	28 (50.9)	68 (37)	
Presence of a family history of autoinflammatory disease, n (%)	10 (18.2)	9 (4.9)	0.001
Presence of multiple site involvement, n (%)	18 (32.7)	31 (16.8)	0.010
Total number of hospitalizations, median (IQR)	2 ( 1‒3 )	1 (1‒2)	0.043
Total length of hospital stay, median (IQR)	27.5 (12‒63.5)	20 (11.5‒34.5)	0.046
Presence of autoinflammatory disease, n (%)	5 (9.1)	4 (2.2)	0.018
Application of topical treatment alone, n (%)	1 (1.8)	37 (20.1)	0.001
Systemic steroids, n (%)			
Systemic steroid alone	16 (35.6)	76 (55.9)	
Systemic steroid + additional immunosuppressive agents	29 (64.4)	60 (44.1)	0.018
Number of patients with good response to systemic steroids (response rate ≥ 75%), n (%)	12 (26.7)	72 (52.9)	0.002
Presence of biological agent use, n (%)	17 (30.9)	27 (14.7)	0.006

IQR, Interquartile Range; PG, Piyoderma Gangrenosum.

**Table 10:** Comparison of characteristics between patients with early onset (onset age < 30 years) PG and late onset (onset age ≥ 30) PG.

Characteristics	Early Onset PG (n = 42)	Late Onset PG (n = 197)	p
Presence of a family history of autoinflammatory disease, n (%)	7 (16.7)	12 (6.1)	0.021
Head and neck involvement, n (%)	4 (9.5)	4 (2)	0.014
Presence of inflammatory bowel disease, n (%)	12 (28.6)	26 (13.2)	0.013
Presence of autoinflammatory disease, n (%)	7 (16.7)	2 (1)	<0.001
Systemic steroids, n (%)			0.001
Systemic steroid alone	9 (25.7)	83 (56.8)	
Systemic steroid + additional immunosuppressive agents	26 (74.3)	63 (43.2)	
Number of patients with good response to systemic steroids (response rate ≥75), n (%)	8 (22.9)	76 (52.1)	0.002
Presence of biological agent use, n (%)	16 (38.1)	28 (14.2)	<0.001

PG, Pyoderma Gangrenosum.

## Discussion

Upon examining comorbidities associated with PG, we found that 51.5% of the patients had at least one comorbidity. The most common comorbidities in the patients were inflammatory arthritis, IBD, HS, hematologic disorders, and solid malignancies respectively. Likewise, in a multicenter study by Ashchyan et al., data from 356 patients with PG were examined, and it was determined that 66.9% of the patients had associated comorbidities.^13^ IBD was found in 41%, inflammatory arthritis in 20.5%, solid organ malignant neoplasms in 6.5%, hematologic malignant neoplasms in 5.9%, other hematologic disorders in 4.8%, and HS in 6.2%.^13^ In the study by Langan et al., PG-associated diseases were detected in 33% of the patients. The most common associated disease was IBD, seen in 20.2% of the cases followed by rheumatoid arthritis (11.8%) and hematologic disorders (3.9%).^14^ Autoinflammatory syndromes (PAPA, PASH, PAPASH) were found in 3.8% of the patients. Evaluating the characteristics of these patients, it was notable that they were generally young and male, had an earlier age of disease onset and had a family history of autoinflammatory diseases.

The most common laboratory finding in the patients was the elevation of CRP (C-reactive protein) levels, seen in 69.7%. Anemia was present in 51.9% of the patients. In a study, elevated CRP levels were found in 56.3% of the patients with PG.^15^ In another study, anemia was seen in 45.6%.^16^ The presence of microbial growth in wound cultures in 46.4% of the patients suggests that PG lesions are prone to secondary infections.

Pathergy was identified in 23% of the patients, while the pathergy status was unknown in 18.4%. Similar to the present study, in the study by Binus et al., pathergy was recorded at a rate of 31.1%;^17^ in the study by Ashchyan et al., the rate was 28.1%.^13^

The most common PG subtype in the patients was ulcerative, accounting for 85% of the cases followed by bullous (4.2%) and pustular (3.8%) PG. In the study by Schosler et al.,^15^ the distribution of PG subtypes was 85.9% ulcerative, 3.1% bullous, 6.3% peristomal, 3.1% vegetative, and 1.6% pustular which was similar to the present study.

The patients receiving systemic treatment were generally treated according to a therapeutic algorithm. Generally, systemic corticosteroids were the initial treatment for the patients. Cyclosporine, azathioprine, dapsone, mycophenolate mofetil, and colchicine were used either as steroid-sparing or adjunctive treatment agents in this study. IVIG and biologicals were used as third-line treatments.

In many studies, systemic steroids are the most commonly used as first-line treatment^15^, ^17^, ^18^ likewise this study. However, additional immunosuppressive treatment is necessary for most of the patients.^18^ When we examined the major characteristics of the group for whom systemic steroids alone were insufficient and additional immunosuppressive treatment was required, we observed that the frequency of the multiple regions’ involvement, multiple lesions, presence of IBD, autoinflammatory diseases, and positive pathergy were higher (all p < 0.05) in this group. The striking findings of the multivariate regression analysis revealing characteristics of the patients who showed good response to systemic steroids were disease onset age ≥ 30, negative pathergy, absence of leukocytosis, and negative wound culture. Complete response was achieved in 28.7% of the patients who used systemic steroids with a median dose of 48 mg of methylprednisolone equivalent and a median duration of 60 days. Herberger et al. evaluated the treatment responses of PG patients with 121 patients in their multicenter study.^18^ The initial treatment in 99% of these patients was systemic steroids at an average dose of 80 mg. The average systemic steroid treatment lasted 4.3 ± 4.6 months. An initial response to systemic steroids was obtained at a rate of 88%. It was reported that patients receiving PG treatment received an average of 2 different systemic treatments and the importance of combination treatment regimens in PG was emphasized.^18^

In the patients, another frequently utilized treatment agent was cyclosporine, administered over a median duration of 100 days and at a dose of 300 mg. Cyclosporine utilized in 35.6% of the patients, achieved a complete response rate of 22.4%. In a multi-center randomized controlled trial comparing systemic steroids and cyclosporine in PG treatment, one group received 0.75 mg/kg/day of prednisolone monotherapy while the other group received 4 mg/kg/day of cyclosporine.^19^ At the end of the 6^th^ week, no difference was observed between the two groups regarding healing rate, treatment response, pain, and relapse duration. However, in the 6^th^ month, healing was not observed in approximately half of the patients in both groups.^19^

Azathioprine was used in 12.6% of the patients. Among the patients who used azathioprine, 16.7% had complete healing. Azathioprine can be used as a steroid-sparing agent in refractory PG or as an alternative to first-line treatments. It may also be a good option in PG accompanied by IBD. Azathioprine generally emerges as a well-tolerated and effective treatment option.^20^ In the present study, azathioprine was also generally used in patients with IBD.

Dapsone was used in 7.9% of the patients. The data regarding the use of dapsone in PG is based on case reports and case series. In the study by Din et al., the treatment responses of 27 patients who received dapsone therapy for PG were evaluated, with 16% of the patients having a complete response and 81% having a partial response.^21^ In the present study, a good response was obtained in 52.6% of the patients.

Colchicine was used in 7.1% of the patients. Few cases have been reported regarding the use of colchicine in PG. Kontochristopoulos et al. reported rapid regression in 2 patients with refractory PG treated with low-dose colchicine monotherapy.^22^ Complete healing was observed in 5.9% of the patients.

Nine of the patients used methotrexate. Among the patients, 77.7% achieved a healing response below 50%. Methotrexate is an immunomodulator and information regarding its use in PG is based on case reports.^23^

Mycophenolate mofetil was administered in 6 of the patients at a median dose of 1.5 gr/day. However, a treatment response below 50% was obtained in all patients. Mycophenolate mofetil is considered a second-line steroid-sparing agent in patients with PG.^20^

In the present study, 10 patients received IVIG treatment. All patients received 1‒3 cycles of treatment at a dose of 2 gr/kg/4-weeks. A treatment response of 75% and above was achieved in 30% of the patients. In the study by Haag et al., they compiled the efficacy of IVIG treatment in refractory cases, identifying complete healing in 23 of 45 patients, and partial response or non-responsiveness in 22 patients.^24^

Biologic agents were used in 18.4% of the patients in this study. Describing the fundamental characteristics of the group necessitating the transition from conventional treatments to biological agent therapy; these included patients with early disease onset age, young patients, males, those with autoinflammatory diseases, those with IBD, those accompanied by HS, and those who were pathergy positive. The median transition time from conventional treatment to biological treatment in the patients was 24-months. Infliximab, the most commonly used biologic agent, was used in 15.1% of the patients. Infliximab was administered to patients at the licensed dose used in psoriasis. The median treatment duration was 12-months and good response was obtained in 63.9% of the patients. Adalimumab was the second most commonly used biologic agent in the patients, used in 7.9% of them. Adalimumab was administered at three different doses: the licensed dose used in psoriasis, 40 mg/week, and 80 mg/week. The average treatment duration was 14-months and the rate of patients responding with ≥ 75% was found to be 5.2%. There were 3 patients who used etanercept. Patients used the licensed dose for an average of 8 months, yet the treatment response did not exceed 50%. Infliximab is the only anti-TNF agent whose efficacy has been demonstrated in classic PG through a randomized double-blind controlled study. In the randomized placebo-controlled study conducted by Brooklyn et al., a partial response was achieved in 48% and a complete response in 21% at the end of 6 weeks.^25^ In phase 3, open-label, multicenter study, the efficacy and safety of adalimumab in refractory PG were investigated, and complete healing was recorded in 54.5% after 26-weeks of treatment.^26^ Abdallah et al. conducted a semi-systematic review concerning the use of TNF-α inhibitors in PG. No difference was found between infliximab, adalimumab, and etanercept in terms of partial and complete response rates.^27^ Especially infliximab and adalimumab from TNF-α antagonists seem to be a good option for resistant cases or to escape the long-term side effects of systemic steroids or cyclosporine.^27^

In the present study, there were 4 patients using ustekinumab with a response rate of 25%‒75%. In a semi-systematic review, the complete response rate was found to be 71% in patients treated with ustekinumab.^28^

IL-1 inhibitors used in PG treatment are anakinra and canakinumab. There were 3 patients using anakinra in the present study. The autoinflammatory disease was present in these patients. Only one of the patients achieved a good response. There was 1 patient using canakinumab in this study who continued treatment for 36 months and responded to the treatment at a rate of ≥75% healing. In the semi-systematic review of Abdallah et al. a complete response was achieved in 57% of the patients (95% CI 45%‒68%).^28^

There were 3 patients using secukinumab in this study. While 2 patients responded at a healing rate of 25%‒50%, 1 patient responded at a healing rate of ≥75%. There was 1 patient receiving risankizumab treatment. A healing response of 75% and above was obtained at the end of 4 months of treatment. There was 1 patient receiving ixekizumab treatment. The treatment was discontinued at the end of 6 months as an adequate response to the treatment could not be obtained. There are case reports indicating that secukinumab, risankizumab, and ixekizumab are effective in resistant PG.^29^, ^30^, ^31^

The present study revealed compelling data concerning two specific patient groups. In patients with pathergy positivity, there was a higher rate of family history of autoinflammatory disease, presence of multiple region involvement, higher total number and total duration of hospitalization, and higher rate of presence of autoinflammatory disease. The pathergy positive group also significantly differed from the pathergy negative group in terms of treatment and treatment responses. In the pathergy positive group, the response rate to systemic steroids was significantly lower, the rate of using adjuvant systemic immunosuppressants was high, and the rate of using biological agents was high. Another specific group was early-onset (onset age < 30) PG. In that group, family history of autoinflammatory disease, head-neck involvement, IBD, and the presence of autoinflammatory disease were at a higher rate. Moreover, in early-onset PG, the rate of patients responding to systemic steroids was low, and the rates of using adjuvant immunosuppressive and biological treatments were significantly high. We believe it is necessary to consider that patients with pathergy positivity and early-onset PG may be more resistant to treatment, requiring more complicated treatments. Indeed, it is crucial to investigate coexisting autoinflammatory diseases at the time of diagnosis in pathergy positive and early-onset PG.

A limitation of the present study is the absence of standard diagnostic criteria for PG which makes it uncertain whether the diagnosis was accurately made in every case. Additionally, we believe that some data might have been lost as this study encompasses patients from the last 10 years. Due to the lack of any scale to score disease severity, response rates were based on physicians’ assessment in the patient records.

## Conclusions

In conclusion, PG is a rare chronic disease accompanied by many comorbidities. While systemic steroids were sufficient in about half of the patients, additional immunosuppressive treatments were necessary in the other half. The presence of multiple lesions, multiple region involvement, autoinflammatory disease and IBD were common characteristics of the patients requiring both additional immunosuppressive and biological treatments. Moreover, we believe that the presence of pathergy positivity and early-onset PG can provide physicians with insight regarding the disease course and prognosis and switching to advanced treatments earlier in these cases might be beneficial. We believe that the data we have compiled from this multi-centered study, which encompasses 10-year data from 12 tertiary care hospitals in the studied country, will contribute to the literature.

## Financial support

None declared.

## Authors’ contributions

Funda Erduran: Conceptualization, data collection, data analysis and interpretation; Writing of the manuscript, critical revision of the article, final approval of the manuscript.

Esra Adışen: Conceptualization, data collection, supervision, critical review and editing, final approval of the manuscript.

Yıldız Hayran: Data collection, data analysis and interpretation, final approval of the manuscript.

Güneş Gür Aksoy: Data collection, editing, final approval of the manuscript.

Erkan Alpsoy: Data collection, editing, final approval of the manuscript.

Leyla Baykal Selçuk: Data collection, editing, final approval of the manuscript.

Sibel Doğan Günaydın: Data collection, editing, final approval of the manuscript.

Ayça Cordan Yazıcı: Data collection, editing, final approval of the manuscript.

Ayşe Öktem: Data collection, editing, final approval of the manuscript.

Malik Güngör: Data collection, editing, final approval of the manuscript.

Elif Afacan: Data collection, editing, final approval of the manuscript.

Deniz Devrim Kuşçu: Data collection, editing, final approval of the manuscript.

Leyla Elmas: Data collection, editing, final approval of the manuscript.

Kübra Aydoğan: Data collection, editing, final approval of the manuscript.

Dilek Bayramgürler: Data collection, editing, final approval of the manuscript.

Evren Odyakmaz Demirsoy: Data collection, editing, final approval of the manuscript.

Melih Akyol: Data collection, editing, final approval of the manuscript.

Rukiye Yasak Güner: Data collection, editing, final approval of the manuscript.

Hilal Kaya Erdoğan: Data collection, editing, final approval of the manuscript.

Ersoy Acer: Data collection, editing, final approval of the manuscript.

Tulin Ergun, Savaş Yaylı: Data collection, editing, final approval of the manuscript.

Ferhan Bulut: Data collection, editing, final approval of the manuscript.

Esra Saraç: Data collection, editing, final approval of the manuscript.

Akın Aktaş: Data collection, editing, final approval of the manuscript.

## Conflicts of interest

None declared.
